# Influenza A and B Virus-Triggered Epithelial–Mesenchymal Transition Is Relevant to the Binding Ability of NA to Latent TGF-β

**DOI:** 10.3389/fmicb.2022.841462

**Published:** 2022-02-25

**Authors:** Wenxian Yang, Xiaoyuan Bai, Heqiao Li, Huizi Li, Wenhui Fan, He Zhang, Wenjun Liu, Lei Sun

**Affiliations:** ^1^CAS Key Laboratory of Pathogenic Microbiology and Immunology, Institute of Microbiology, Chinese Academy of Sciences, Beijing, China; ^2^Savaid Medical School, University of Chinese Academy of Sciences, Beijing, China; ^3^Institute of Infectious Diseases, Shenzhen Bay Laboratory, Guangdong, China; ^4^Institute of Microbiology, Center for Biosafety Mega-Science, Chinese Academy of Sciences, Beijing, China

**Keywords:** influenza A virus, influenza B virus, epithelial-mesenchymal transition, neuraminidase, transforming growth factor-beta, pulmonary fibrosis

## Abstract

Epithelial–mesenchymal transition (EMT) is an important mechanism of lung tissue repair after injury, but excessive EMT may lead to pulmonary fibrosis, respiratory failure, and even death. The EMT triggered by influenza A virus (IAV) and influenza B virus (IBV) is not well understood. We hypothesized that there was difference in EMT induced by different influenza virus strains. Here we discovered that both IAV [A/WSN/1933 (H1N1), WSN] and IBV (B/Yamagata/16/88, Yamagata) infection caused EMT in mouse lung and A549 cells, and more EMT-related genes were detected in mice and cells infected with WSN than those infected with Yamagata. Neuraminidase (NA) of IAV is able to activate latent TGF-β and the downstream TGF-β signaling pathway, which play a vital role in EMT. We observed that IAV (WSN) triggered more activated TGF-β expression and stronger TGF-β/smad2 signaling pathway than IBV (Yamagata). Most importantly, WSN NA combined more latent TGF-β than Yamagata NA in A549 cells. Collectively, these data demonstrate that both IAV and IBV induce TGF-β/smad2 signaling pathway to promote EMT, which might depend on the binding ability of NA to latent TGF-β.

## Introduction

Influenza virus infection will lead to acute and severe respiratory diseases (Donaldson et al., 2009; Gaitonde et al., 2019; Liu et al., 2021) and be accompanied by other common complications such as acute respiratory distress syndrome (Chow et al., 2019), secondary bacterial pneumonia (Estenssoro et al., 2010; Farias et al., 2010; Rice et al., 2012; Bai et al., 2021), and pulmonary fibrosis (Qiao et al., 2009; Shatskaya et al., 2017). Epithelial–mesenchymal transition (EMT) is an important mechanism of lung tissue repair (Duffield et al., 2013; Jolly et al., 2018), but excessive EMT may lead to pulmonary fibrosis, respiratory failure, and even death (Lv et al., 2020; Peng et al., 2020; Phan et al., 2021). TGF-β is the most common cytokine that induces EMT. Unlike most cytokines, TGF-β is secreted by virtually all cells in a biologically inactive form termed latent TGF-β. Latent TGF-β is composed of an amino-terminal latency-associated peptide (LAP) that remains non-covalently associated with the carboxy-terminal mature TGF-β molecule (Ballesteros et al., 2015). The release of mature TGF-β from the LAP is necessary to bind to cellular receptors and activate the TGF-β/smad2/3 signaling pathway, leading to alveolar epithelial cells changing from cuboidal to an elongated spindle shape (Gonzalez and Medici, 2014; Lamouille et al., 2014; Yao et al., 2019).

Upon influenza virus infection, neuraminidase (NA) interacts with latent TGF-β, resulting in activation, possibly through cleavage of the sialic acid residues on latent TGF-β. It has been reported that most subtypes of influenza A virus (IAV) (H3N8, H12N5, and H7N7), including human strains (H1N1 and H2N2) and influenza B virus (IBV) (B/David Breeze), increase TGF-β activity in the chemically defined or cell systems (Schultz-Cherry and Hinshaw, 1996; Carlson et al., 2010; Li et al., 2015). Considering the diversity of NA sequence among influenza virus strains and the role of NA in TGF-β activation, we speculated that there might be a difference in EMT upon different IAV and IBV stain infection.

In the present study, we compare the EMT triggered by IAV (WSN) and IBV (Yamagata) and find that WSN induces stronger EMT than Yamagata, which is relevant to the binding ability of NA to latent TGF-β.

## Materials and Methods

### Mice

BALB/c mice were purchased from Jackson Laboratory and fed in the animal facility of the Institute of Microbiology. Mice were maintained under specific pathogen-free (SPF) conditions.

### Ethics Statement

All animal experiments were approved by the Research Ethics Committee of the Chinese Academy of Sciences and complied with the Beijing Laboratory Animal Welfare and Ethical Guidelines of the Beijing Administration Committee of Laboratory Animals. All mice were maintained in a barrier facility with free access to food and water.

### Cell Culture

A549 cell line, derived from human alveolar epithelial carcinoma, was obtained from American Type Culture Collection (CCL185). Cells were maintained in DMEM (Invitrogen) medium containing 10% FBS and 1% antibiotics.

### Virus and Virus Titration

Human influenza A strain H1N1 (WSN) and B stain (Yamagata) were used and maintained in MDCK cells. Virus infectivity was assessed by titration in MDCK cells by plaque assay. Briefly, the MDCK cells were infected at confluency by serial dilutions of virus and incubated for 1 h at 37°C and 5% CO_2_. After 1 h of adsorption, the inoculum was removed, and cells were incubated with MEM with noble agar (0.65%) and 2 μg/ml of L-1-tosylamide-2-phenylethyl chloromethyl ketone-treated trypsin (TPCK trypsin, Sigma) for 48 h. Finally, the lytic plaques were counted by staining with violet crystal after 48 h post-infection.

### Virus Infection *in vivo*

Eight-week-old female or male BALB/c mice were anesthetized and inoculated intranasally with IAV (WNS) or IBV (Yamagata) at PFU = 3000 in 30 μl of PBS.

### Masson’s Trichrome Staining of Lung Sections

Formalin-fixed lung tissue was embedded in paraffin, and 5-μm sections were stained with Masson’s trichrome reagent to demonstrate collagen. The procedure was as follows: (i) fix in Bouin’s or Zenker’s liquor for one night; (ii) wash in running water until the yellow color disappears and rinse in two changes of distilled water; (iii) stain with Mayer’s hematoxylin for 5 min; (iv) place in 0.5% hydrochloric acid in 70% alcohol for 5 s; (v) wash in running tap water for 30 s and rinse in two changes of distilled water; (vi) stain with acid ponceau for 5–10 min; (vii) rinse in three changes of distilled water; (viii) dissolve in 1% phosphomolybdic aqueous acid solution; (ix) stain with aniline blue or brilliant green for 5 min; (x) dissolve in 1% glacial acetic acid for 5 min; (xi) dehydrate in 95% ethyl alcohol several times, followed by anhydrous alcohol; (xii) hyalinize with dimethyl benzene; and (xiii) seal with neutral balsam. With the stain, collagen fibers are stained blue; the cytoplasm, muscle fibers, and red blood cells are stained red; and the nuclei are stained black.

### Virus Infection *in vitro*

A549 cells were grown in 12-well plates and incubated for 2 h in medium DMEM without FBS prior treatments conditions. A549 cells were infected with the virus at a multiplicity of infection (MOI) of 0–0.01, according to the experiment.

### Trypan Blue Staining

Trypan blue staining was used at different times post-infection to observe the cellular death. The 0.4% Trypan blue was treated for about 2 min; then, the number of Trypan blue-positive cells was calculated by counting at least three random separate fields.

### Cell Migration Assay

In the migration experiment, 4 × 10^4^ A549 cells, in serum-free medium, were seeded into the upper chamber of a Transwell insert (8-mm pore size; Corning Inc.) and infected by WSN or Yamagata, respectively; meanwhile, a medium with 20% FBS was added in the lower chamber as a chemoattractant. After incubation at 37°C, 5% CO_2_ for 48 h, the Transwell chamber was taken out, and the medium in the well was discarded and washed with calcium-free PBS. Then, the cells were fixed with methanol for 30 min and stained with 0.1% crystal violet for 20 min. The upper un-migrated cells were gently wiped off with a cotton swab and counted under a microscope.

### Western Blot Assay

At different times post-infection, the protein fraction of A549 cells were extracted by using the lysis buffer [25 mM Tris/HCl, pH 7.5, 150 mM NaCl, 10% glycerol, 0.1% SDS, 0.5% sodium deoxycholate, 1% IgePal, 2 mM EDTA, 2 mM EGTA, pH 8.0, complemented with proteases (Roche, Basel, Switzerland) and phosphatases inhibitors cocktail (Roche, Basel, Switzerland)], on ice for a minimum of 30 min. Supernatants were cleared by centrifugation in a standard tabletop centrifuge (Labnet) at maximum speed (13,000 rpm) for 15 min. Protein concentration was determined by Bradford assay (Bio-Rad, Hercules, CA, United States), and 25 μg of protein was loaded per lane into reduced SDS-PAGE gels of different percentages. After separation, proteins were transferred to a nitrocellulose membrane for Western blotting. Nitrocellulose membranes were blocked with 1% BSA or 5% of fat-free dry milk by 2 h at room temperature and probed for SMAD7 (goat IgG N-19 clone, Santa Cruz Biotech, Dallas, Texas, United States), SMAD2/3 (goat IgG N-19 clone, Santa Cruz Biotech, Dallas, Texas, United States), phospho-SMAD 2/3 (rabbit IgG Ser 423/425, Santa Cruz Biotech, Dallas, Texas, United States), EMT Antibody Sampler Kit (rabbit monoclonal antibody, CST, Cat#9782T), and Wnt/β-Catenin Activated Targets Antibody Sampler Kit (rabbit monoclonal antibody, CST, Cat #8655T), followed by horseradish peroxidase-conjugated immunoglobulin secondary antibody (Jackson ImmunoResearch). Immune complexes were detected by enhanced chemiluminescence (Perkin Elmer, Waltham, MA, United States) and recorded by ChemiDoc XRS (Bio-Rad, Hercules, CA, United States).

### Coimmunoprecipitation Assay

Target plasmids were transfected into A549 cells with Lipofectamine 2000 (Thermo Fisher Scientific) for 24 h and adequately lysed using a lysis buffer containing 0.5% NP-40, 150 mM NaCl, 20 mM HEPES (pH 7.4), 10% glycerol, and 1 mM EDTA with complete protease inhibitor cocktail and PhosSTOP™ phosphatase inhibitor tablets (Roche). The mixture was then combined with anti-c-Myc agarose affinity gel, ANTI-FLAG^®^ M2 affinity gel for 4–6 h. After combination, the beads were washed four to five times with washing buffer (300 mM NaCl, 20 mM HEPES, 1 mM EDTA, 1% Triton X-100, and 10% glycerin), then the bound proteins were eluted by boiling for 10 min in SDS protein loading buffer and analyzed by immunoblot with mouse monoclonal anti-FLAG M2, mouse monoclonal anti-c-Myc, and mouse monoclonal anti-HA, followed by the incubation with HRP goat anti-mouse/rabbit IgG secondary antibodies.

### RNA Extraction, cDNA Synthesis, and qPCR Analysis

According to the manufacturer’s instructions, total RNA from A549 cells and lung tissue homogenate with TRIzol (Invitrogen). The “gDNA eliminator” column was applied to remove genomic DNA in all samples. RNA quantity and quality were measured using the Agilent 2100 Bioanalyzer for each experiment. The RNA with a high RNA integrity Number (RIN) (≥9) was used for cDNA preparation prior to quantitative real-time PCR experiments.

According to the manufacturer’s instructions, cDNA was synthesized from 1 μg of total RNA using an oligo (dT) primer and M-MLV reverse transcriptase (Promega). To ensure the removal of genomic DNA, “gDNA wipe-out buffer” was added to RNA prior to the RNA conversion step. Relative gene expression was analyzed by qPCR using SYBR Premix Ex Taq (TaKaRa). The primers are listed in Table 1. The amplification process involves 45 cycles of 95^°^C for 10 s followed by 60^°^C for 10 s and finally 72^°^C for 10 s. The Ct values generated from an ABI 7500 were analyzed using the 2^–ΔΔCt^ method. The expression of target genes was normalized to that of GAPDH.

**TABLE 1:** Primers used in this study.

Genes	Primers	Sequence (5’→3’)
*TGFB1*	Forward	AGCTGTACCAGAAATACAGCA
	Reverse	ATAACCACTCTGGCGAGTC
*CDH1*	Forward	CAGAATGACAACAAGCCCGAA
	Reverse	TGAGGATGGTGTAAGCGATGG
*CDH2*	Forward	AGGCAGAAGAGAGACTGGGT
	Reverse	GCTGTACCGCAGAGAAAGGT
*CTNNB1*	Forward	GCTCTTGTGCGTACTGTCCT
	Reverse	GTCCGTAGTGAAGGCGAACA
*SNAI1*	Forward	CCGGAGACCTAGATGTCATTGT
	Reverse	CCGTCTGGGAATCACTGTCC
*PPARG*	Forward	TACTGTCGGTTTCAGAAATGCC
	Reverse	GTCAGCGGACTCTGGATTCAG
*VIM*	Forward	AGTCCACTGAGTACCGGAGAC
	Reverse	CATTTCACGCATCTGGCGTTC
*CCND1*	Forward	TGGAGCCCGTGAAAAAGAGC
	Reverse	TCTCCTTCATCTTAGAGGCCAC
*TCF1*	Forward	CAGAAGCAAGTTCACAGGC
	Reverse	TAGCATCAAGGATGGGTGG
GAPDH	Reverse	TTGTCTCCTGCGACTTCAACG
	Reverse	GGTCTGGGATGGAAATTGTGAG
*Tgfb1*	Forward	ACCAAGGAGACGGAATACAG
	Reverse	CGTTGATTTCCACGTGGAG
*Cdh1*	Forward	TCAGTTCCGAGGTCTACAC
	Reverse	CTTCAAATCTCACTCTGCCC
*Cdh2*	Forward	GGACACGAATGACATATAAAGG
	Reverse	CCCTACACTAAACACCAAGAC
*Ctnnb1*	Forward	CGCCTTCATTATGGACTGC
	Reverse	TCCAACAGTTGCCTTTATCAG
*Snai1*	Forward	GAAGATGCACATCCGAAGC
	Reverse	GAATGGCTTCTCACCAGTG
*Pparg*	Forward	GATGTCTCACAATGCCATCAG
	Reverse	ATATCACTGGAGATCTCCGC
*Vim*	Forward	CCCTTAAAGGCACTAACGAG
	Reverse	GGTAGTTAGCAGCTTCAAGG
*Ccnd1*	Forward	AGACCATTCCCTTGACTGC
	Reverse	AAGCAGTTCCATTTGCAGC
*Tcf1*	Forward	CCAAGGTCATTGCTGAGTG
	Reverse	GAGATAGTGCATGCCACCT
*Gapdh*	Forward	ACTCTTCCACCTTCGATGC
	Reverse	CCGTATTCATTGTCATACCAGG

### Statistical Analysis

Statistical analyses were performed using GraphPad Prism 9 software and Microsoft Excel. Data are presented as the mean values ± SD of at least three independent experiments. Comparisons between two groups were performed using the two-tailed Student’s *t*-test. *p* < 0.05 was considered significant, with *p < 0.05 or ^**^p < 0.01.

## Results

### Influenza A Virus and Influenza B Virus Trigger Epithelial–Mesenchymal Transition-Mediated Pulmonary Fibrosis in Mice

**FIGURE 1:**
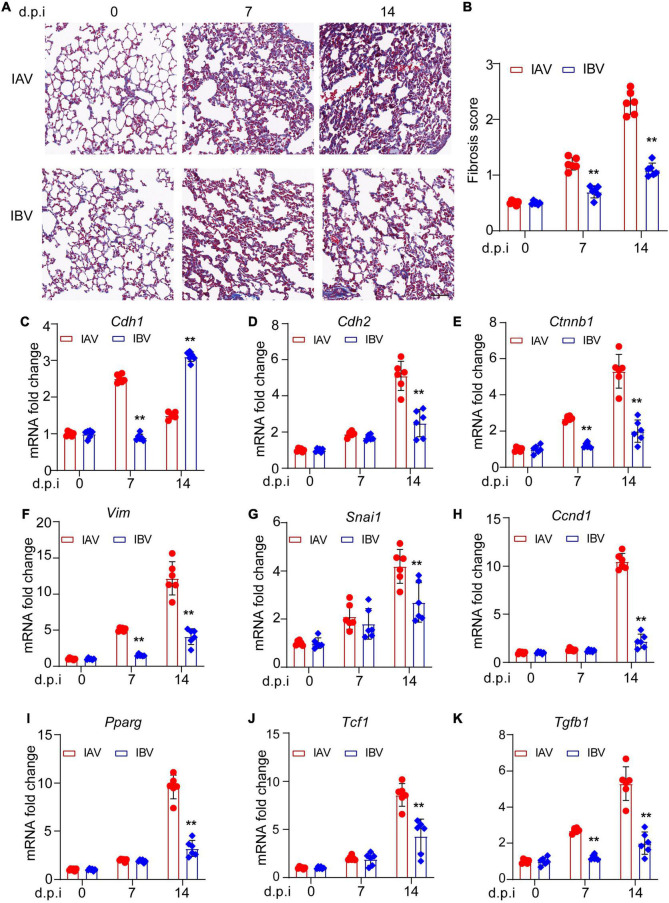
Influenza A and B viruses induce pulmonary fibrosis and EMT-related gene expression in mice. Balb/c mice were intranasally treated with IAV (WSN) or IBV (Yamagata) (PFU = 3000) for the indicated time points. **(A)** Masson staining of lungs. Scale bar, 200 μm. **(B)** Fibrosis scores. qPCR analysis of E-cadherin **(C)**, N-cadherin **(D)**, β-catenin **(E)**, vimentin **(F)**, snail **(G)**, cyclin D **(H)**, PPAR **(I)**, TCF-1 **(J)**, and TGF-β **(K)** in mouse lungs. Data are representative of three independent experiments (*n* = 6). Data are presented as the mean ± SD for panels **(B–K)**. **p < 0.01 (unpaired, two-tailed Student’s *t*-test).

There are very few studies about influenza virus-induced pulmonary fibrosis (Qiao et al., 2009; Shatskaya et al., 2017; Huang and Tang, 2021). It has been reported that IAV (influenza A/H1N1 A/Tomsk/13/2010) induces EMT-mediated pulmonary fibrosis in mice (Shatskaya et al., 2017). However, whether other strains of IAV or IBV could also trigger EMT and whether there was difference in EMT upon different influenza virus strain infection is still unknown. The pulmonary fibrosis was investigated in IAV [A/WSN/1933 (H1N1), WSN]- and IBV (B/Yamagata/16/88, Yamagata)-infected mice. The Masson’s trichrome staining results showed that WSN induced more fibrosis than Yamagata at Days 7 and 14 post-infection (Figure 1A) and fibrosis scoring (Figure 1B). Hallmarks of EMT include the loss of expression or function of E-cadherin and reduced abundance of tight junction proteins (Gonzalez and Medici, 2014; Lamouille et al., 2014). We observed that the expression of E-cadherin, the epithelial marker, was lower in lungs of mice upon WSN infection than that upon Yamagata infection (Figure 1C), while the expression of mesenchymal markers, such as N-cadherin (Figure 1D), β-catenin (Figure 1E), vimentin (Figure 1F), snail (Figure 1G), cyclin D (Figure 1H), PPAR (Figure 1I), TCF-1 (Figure 1J), and TGF-β (Figure 1K), was significantly higher in lungs of mice upon WSN infection than that upon Yamagata infection. These data demonstrate that both IAV (WSN) and IBV (Yamagata) infection cause EMT in mouse lung, and more EMT-related genes are detected in mice infected with WSN than those infected with Yamagata.

### Influenza A Virus and Influenza B Virus Induce the Epithelial–Mesenchymal Transition of A549 Cells

**FIGURE 2:**
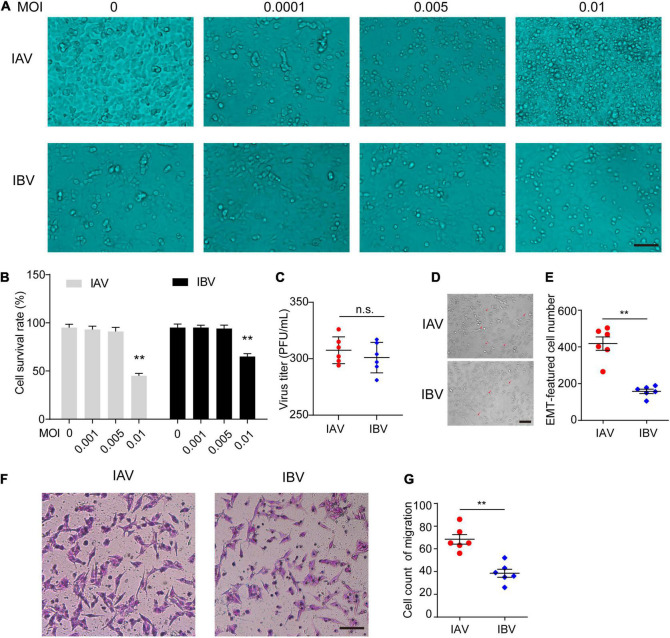
Influenza A and B viruses induce the EMT of A549 cells. **(A)** Images acquired with a microscope of A549 infected with different dose of IAV (WSN) or IBV (Yamagata) for 48 h. Scale bar, 50 μm. **(B)** The survival rate of A549 cells stained by Trypan blue in **(A)** (**p < 0.01 vs. MOI = 0). **(C)** The virus titers of WSN and Yamagata (MOI = 0.005) at 48 h (h) post-infection in A549 cells. **(D)** Images of A549-EMT morphology induced by WSN or Yamagata (MOI = 0.005) for 48 h. **(E)** Cell counts of EMT-featured A549 cells of each well in **(D)**. **(F)** Cell migration assays in A549 cells stimulated with WSN or Yamagata (MOI = 0.005) for 48 h. Scale bar, 100 μm. **(G)** Cell counts of migrated A549 cells infected by WSN or Yamagata. Data are representative of three independent experiments (*n* = 6). Data are presented as the mean ± SD for panels **(B,D,F)**. n.s.: not significant. **P < 0.01 (unpaired, two-tailed Student’s *t*-test).

We further confirmed these findings *in vitro*. The optimum MOI of WSN and Yamagata for inducing EMT was first determined in A549 cells. As a result, WSN and Yamagata induced EMT without mass cell death at an MOI of 0.005 (Figures 2A,B). Moreover, the virus titers had no significant difference between WSN and Yamagata (MOI = 0.005) at 48 h post-infection in A549 cells (Figure 2C). Subsequently, A549 cells were infected with WSN and Yamagata at an MOI of 0.005, respectively, and the numbers of spindle cells that are the feature of EMT were counted after 48 h post-infection. WSN induced more EMT-featured A549 cells than Yamagata (Figures 2D,E). Meanwhile, the results of the cell migration assay showed that the cell migration was stronger after WSN infection than that after Yamagata infection (Figures 2F,G). These results indicate that IAV (WSN) induces more EMT of A549 cells than IBV (Yamagata).

### Influenza A Virus and Influenza B Virus Enhance the Expression of Epithelial–Mesenchymal Transition-Related Genes in A549 Cells

**FIGURE 3:**
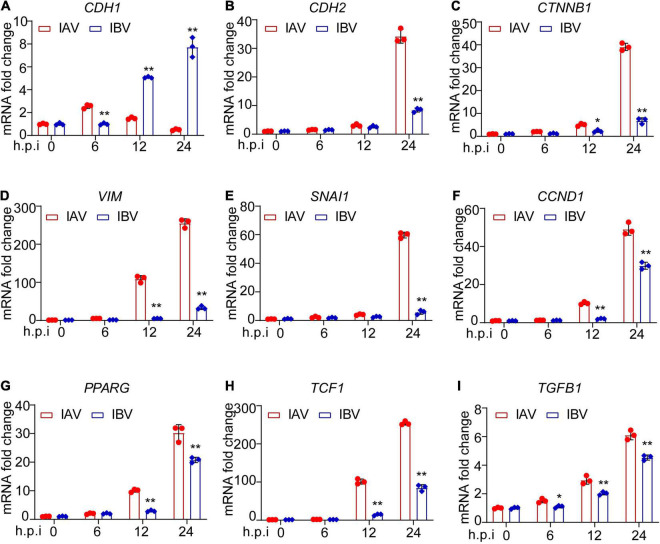
Influenza A and B viruses enhance the mRNA expression levels of EMT-related genes in A549 cells. A549 cells were infected by IAV and IBV (MOI = 0.005) for 0, 6, 12, and 24 h and the expression of E-cadherin **(A)**, N-cadherin **(B)**, β-catenin **(C)**, vimentin **(D)**, snail **(E)**, cyclin D **(F)**, PPAR **(G)**, TCF-1 **(H)**, and TGF-β **(I)** was detected by qPCR. Data are representative of three independent experiments (*n* = 3). Data are presented as the mean ± SD. *p < 0.05 and **p < 0.01 (unpaired, two-tailed Student’s *t*-test).

**FIGURE 4:**
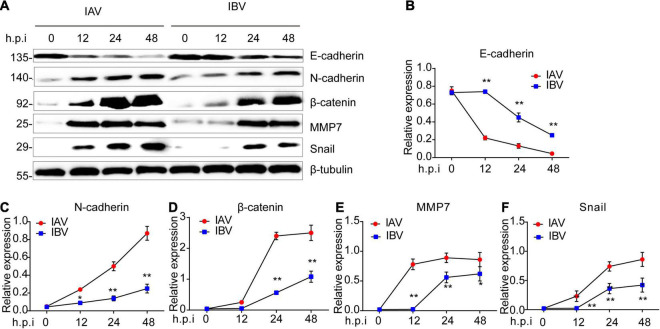
Influenza A and B viruses trigger the protein expression levels of EMT-related genes in A549 cells. **(A)** Immunoblot analysis of EMT-relevant proteins in A549 infected with IAV (WSN) and IBV (Yamagata) (MOI = 0.005) for the indicated time-points. The relative expression of E-cadherin **(B)**, N-cadherin **(C)**, β-catenin **(D)**, MMP7 **(E)**, and Snail **(F)** in **(A)**. Data are representative of three independent experiments. Data are presented as the mean ± SD for panels **(B–E)**. *p < 0.05 and **p < 0.01 (unpaired, two-tailed Student’s *t*-test).

The mRNA expression level of EMT-related genes was investigated in A549 cells infected with influenza virus by using qPCR. It was observed that the mRNA expression level of E-cadherin was lower at 12 and 24 h after WSN infection than Yamagata infection (Figure 3A). In contrast, WSN induced more N-cadherin (Figure 3B), β-catenin (Figure 3C), vimentin (Figure 3D), snail (Figure 3E), cyclin D (Figure 3F), PPAR (Figure 3G), TCF-1 (Figure 3H), and TGF-β (Figure 3I) expression than Yamagata at 12 and/or 24 h after virus infection. Similar results were observed in the Western blotting assay to measure the protein levels of E-cadherin, N-cadherin, β-catenin, MMP7, and Snail (Figures 4A–F). All these data strongly support the above results.

### Influenza A Virus and Influenza B Virus-Triggered Epithelial–Mesenchymal Transition Is Relevant to NA-Activated TGF-β/Smad2 Signaling Pathway

**FIGURE 5:**
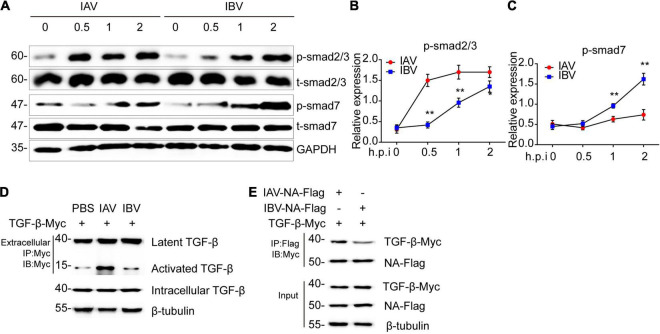
Influenza A and B virus-activated TGF-β/smad2 signaling pathway is relevant to the binding ability of NA to latent TGF-β. **(A)** Immunoblot analysis of the phosphorylation of the indicated proteins in A549 infected with IAV (WSN) and IBV (Yamagata) (MOI = 0.005) for the indicated time-points. The relative expression of p-smad2/3 **(B)** and p-smad7 **(C)** in **(A)**. **(D)** Immunoblot analysis of the indicated proteins in immunoprecipitated samples from supernatant of A549 cells transfected with TGF-β-myc for 24 h and then stimulated by IAV and IBV (MOI = 0.005) for 2 h. **(E)** Immunoblot analysis of the indicated proteins in immunoprecipitated samples of A549 cells transfected with various combinations of plasmids for 24 h. Data are representative of three independent experiments. Data are presented as the mean ± SD for panels **(B,C)**. *p < 0.05 and **p < 0.01 (unpaired, two-tailed Student’s *t*-test).

The TGF-β/smad2 signaling pathway plays an important role in EMT (Gonzalez and Medici, 2014; Ballesteros et al., 2015; Yao et al., 2019). Thus, Western blotting was performed to determine whether IAV (WSN) and IBV (Yamagata) could activate TGF-β/smad2 signaling pathway. After virus infection, higher levels of phosphorylated-smad2/3 and lower levels of smad7 were found in WSN-infected A549 cells than in Yamagata-infected A549 cells (Figures 5A–C), indicating that WSN trigged a stronger TGF-β/smad2 signaling pathway than Yamagata. Additionally, the coimmunoprecipitation assays showed that WSN triggered more activated TGF-β expression than Yamagata in the supernatant of A549 cells (Figure 5D).

It is well established that the neuraminidase (NA) of influenza virus interacts with latent TGF-β, resulting in activation, possibly through cleavage of the sialic acid residues on latent TGF-β (Schultz-Cherry and Hinshaw, 1996; Li et al., 2015). Therefore, we speculated that the difference between WSN-triggered and Yamagata-triggered EMT might depend on the interaction of NA with latent TGF-β. As expected, the coimmunoprecipitation assays showed that WSN NA combined more latent TGF-β than Yamagata NA in A549 cells (Figure 5E). These data suggest that a stronger interaction between NA and latent TGF-β might facilitate the activation of the latent TGF-β and TGF-β/smad2/3 signaling pathway and ultimately induces more EMT. Together, IAV (WSN), and IBV (Yamagata) induce the TGF-β/smad2 signaling pathway to promote EMT, which might depend on the binding ability of NA to TGF-β.

## Discussion

Virus-triggered EMT is believed to contribute to the progression of disease, including pulmonary fibrosis and cancer. Many viruses, such as human papillomavirus (HPV) (Bernard et al., 2010), Epstein-Barr virus (EBV) (Wang et al., 2014; Gaur et al., 2015), hepatitis B and C virus (HBV, HCV) (Yang et al., 2009; Bose et al., 2012), and severe acute respiratory syndrome (SARS)-CoV-2 (Lai et al., 2021) are known to be involved in the EMT process. However, little is known about influenza virus-induced EMT, especially IBV. Although it has been reported that IAV (influenza A/H1N1 A/Tomsk/13/2010) induces EMT-mediated pulmonary fibrosis in mice (Shatskaya et al., 2017), whether other strains of IAV or IBV could also trigger EMT is still unknown. Here we show that both IAV (WSN) and IBV (Yamagata) can induce EMT, leading to pulmonary fibrosis in mice.

After IAV (WSN) and IBV (Yamagata) infection, the expression levels of epithelial and mesenchymal markers are both fluctuated in mouse lung and A549 cells. Specifically, the expression of epithelial marker E-cadherin is decreased and its repressor Snail is increased. Meanwhile, the expression of mesenchymal markers N-cadherin, vimentin, and β-catenin and the downstream of Wnt/β-catenin, cyclin D, PPARγ, and TCF-1 are enhanced. In addition, MMP7 expression was increased. This results in A549 cells changing from cuboidal to an elongated spindle shape, increasing the ability of migration, and final EMT, suggesting that both IAV (WSN) and IBV (Yamagata) can induce the EMT of alveolar epithelial cells. To our knowledge, these are the first studies demonstrating that IBV can trigger EMT.

The progression of EMT is regulated by the crosstalk of multiple signaling pathways, such as TGF-β/smad2/3 (Xu et al., 2009), PI3K/AKT (Yu et al., 2017), Wnt/β-catenin (Tang et al., 2020), ERK/MAPK (Sheng et al., 2020), and p38/MAPK (Wen et al., 2019). Distinct pathways can be activated by one or more factors (Lamouille et al., 2014). It is well documented that the activation of TGF-β by NA protein of IAV is critical for TGF-β/smad2 signaling pathway-mediated EMT (Schultz-Cherry and Hinshaw, 1996; Li et al., 2015; Yao et al., 2019, 2020). Previous studies have demonstrated that most IAV subtypes (H1N1, H1N2, H3N2, H5N9, H6N1, and H7N3) activate latent TGF-β except the highly pathogenic H5N1 strains, and the 19-amino-acid deletion of H5N1 NA contributes to the decreased ability to activate latent TGF-β (Carlson et al., 2010). In the present study, we observe that both IAV (WSN) and IBV (Yamagata) can also activate latent TGF-β and the downstream TGF-β/smad2 signaling pathway. Moreover, NA proteins of WSN and Yamagata interact with latent TGF-β, indicating that the NA-activated TGF-β/smad2 signaling pathway is critical for EMT and pulmonary fibrosis during IAV and IBV infection.

We also discovered the difference in EMT induced by a different influenza virus strain. More EMT-related genes were detected in mice and cells infected with IAV (WSN) than those infected with IBV (Yamagata), and WSN triggered more activated TGF-β expression and a stronger TGF-β/smad2 signaling pathway than Yamagata. Most importantly, WSN NA combined more latent TGF-β than Yamagata NA in A549 cells, which might associate with the low amino acid sequence identity (24.7%) between WSN-NA and Yamagata-NA. Based on these results, we believe that IAV- and IBV-induced EMT might be relevant to the binding ability of NA to TGF-β, which reveal a previously unrecognized mechanism underlying influenza virus-mediated EMT. Certainly, there is the possibility that IAV and IBV induce TGF-β activation and EMT independent of NA, which is interesting and worthy of further study.

In summary, we investigate and compare the EMT process triggered by IAV (WSN) and IBV (Yamagata). The results show that both IAV and IBV can induce EMT, and WSN triggers stronger EMT than Yamagata, which might be relevant to a stronger binding of latent TGF-β to NA of WSN than that of Yamagata, providing the theoretical contribution for understanding the mechanism of influenza virus-triggered EMT. Considering that the IAV and IBV virus strains are limited to WSN and Yamagata in this study, further investigation into the difference in EMT during other types of influenza virus infection is required to confirm the underlying mechanism.

## Data Availability Statement

The original contributions presented in the study are included in the article/supplementary material, further inquiries can be directed to the corresponding author.

## Ethics Statement

The animal study was reviewed and approved by the Research Ethics Committee of the Chinese Academy of Sciences.

## Author Contributions

LS initiated the project and supervised the project. LS and WY designed the experiments, analyzed the data, and wrote the manuscript. WY performed the experiments. XB, HeL, HuL, and HZ helped with some experiments. WL and WF helped analyze the data and revised the manuscript. All authors contributed to the article and approved the submitted version.

## Conflict of Interest

The authors declare that the research was conducted in the absence of any commercial or financial relationships that could be construed as a potential conflict of interest.

## Publisher’s Note

All claims expressed in this article are solely those of the authors and do not necessarily represent those of their affiliated organizations, or those of the publisher, the editors and the reviewers. Any product that may be evaluated in this article, or claim that may be made by its manufacturer, is not guaranteed or endorsed by the publisher.
